# Abdominal Stent Grafting With Coil Embolization for an Abdominal Aortic Aneurysm Sac With a Short Neck

**DOI:** 10.7759/cureus.58988

**Published:** 2024-04-25

**Authors:** Daisuke Sakamoto, Takuya Sakamoto, Yasuhiro Nagayoshi, Tamaki Takano

**Affiliations:** 1 Cardiovascular Surgery, Kanazawa Medical University Hospital, Uchinada, JPN; 2 Medical Research Institute, Kanazawa Medical University, Uchinada, JPN; 3 Cardiovascular Surgery, Kanazawa Medical University, Uchinada, JPN

**Keywords:** coil embolization, endoleak, short neck, endovascular abdominal aortic aneurysm repair, abdominal aortic aneurysm

## Abstract

A 68-year-old man underwent endovascular abdominal aortic aneurysm repair for a two-humped abdominal aortic aneurysm (AAA) with a short neck. The abdominal aorta had severe calcification, suggesting a high risk for type Ia endoleak. Initially, a catheter was placed in the aneurysm sac, followed by stent graft deployment. Then, coils were inserted into the aneurysm neck. Subsequently, the type Ia endoleak was resolved. One year after the surgery, no evidence of endoleak was observed, and the aneurysm size had decreased by 10 mm. Therefore, this procedure may be effective for short-neck AAAs.

## Introduction

Endovascular abdominal aortic aneurysm repair (EVAR) is a common procedure for abdominal aortic aneurysms (AAAs) [1]. However, no procedure has been established for a challenging neck, such as a short neck. Currently, treatments for short-neck AAAs include creating holes in the stent graft body aligned with abdominal branches by using the fenestration method (fenestrated endovascular stent grafts) or placing stents in the renal arteries by using the chimney method. However, both fenestration and chimney methods demonstrate high secondary intervention rates. In this report, we present a case of an AAA with a short neck complicated with endoleak, which was eliminated by inserting coils in the aneurysm neck. This technique may be effective and potentially reliable for AAAs with a challenging neck. This patient provided written informed consent to use his details and imaging studies for our case report.

## Case presentation

**Figure 1:**
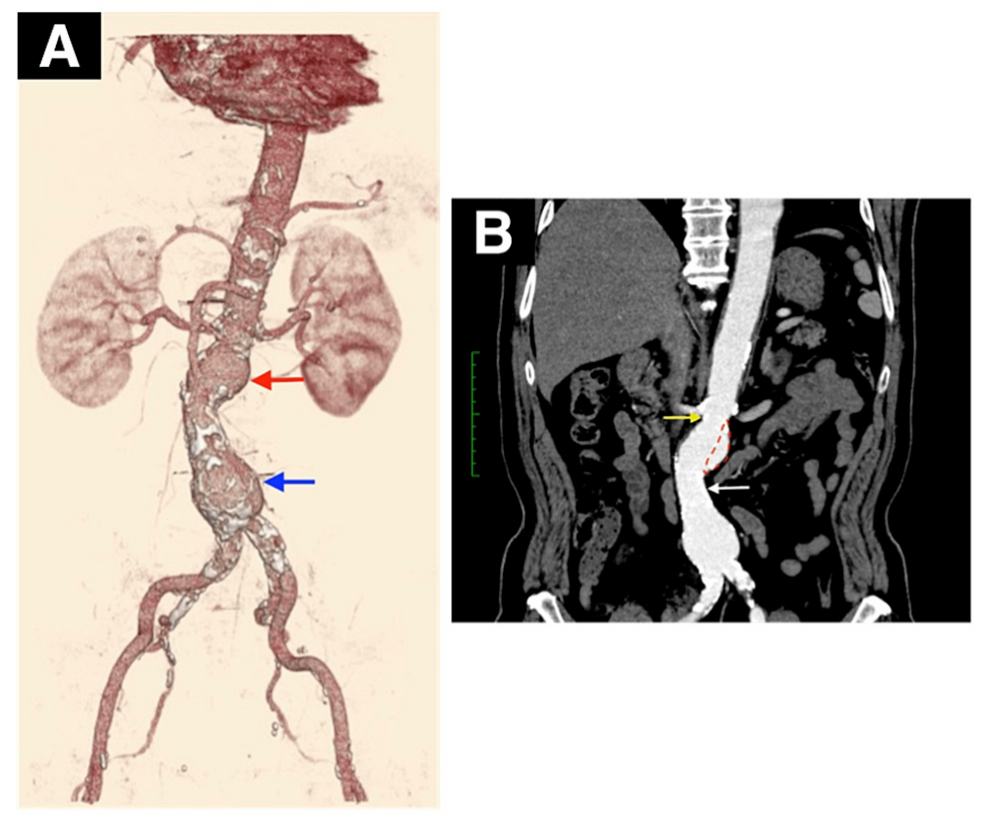
(A) Preoperative three-dimensional reconstruction computed tomography of the abdominal aorta shows severe calcification and multiple abdominal aortic aneurysms.The first aneurysm was 40 mm in diameter (red arrow) and the second aneurysm was 50 mm (blue arrow). (B) The first short neck length was 7 mm (yellow arrow) and second neck length was 30.5 mm (white arrow). The red dotted lines indicate coil embolization areas.

A 68-year-old man with a hypertension history since the age of 60 underwent percutaneous coronary intervention (PCI) for acute myocardial infarction. During this PCI, a computed tomographic (CT) scan revealed the presence of a 45 mm AAA. Six months later, the aneurysm had expanded to 50 mm, necessitating surgical intervention. Considering the patient’s severe obesity (body mass index > 30 kg/m^2^) and dual antiplatelet therapy, open surgical repair (OSR) posed significant risks. The patient preferred a less invasive treatment, and EVAR was chosen. The patient’s blood pressure was 138/60 mmHg, with a pulse rate of 80 beats/minute. Physical examination of the chest and abdomen revealed no abnormalities. Abdominal contrast-enhanced CT scan showed a two-humped AAA (Figure 1A). The neck length from the lowest renal artery to the beginning of the aortic sac was 7 mm, which is considered short. The neck diameter measured 26.5 mm but presented a conical shape. The first aneurysm was 40 mm in diameter. The second neck length was 30.5 mm, with a diameter of 27.0 mm and mild angulation. The diameter of the second aneurysm was 50 mm. Furthermore, the aorta exhibited significant calcification and internal thrombosis. The left and right renal arteries branched at nearly the same level, with a distance of 8 mm between the superior mesenteric artery and the renal arteries (Figure 1B).

**Figure 2:**
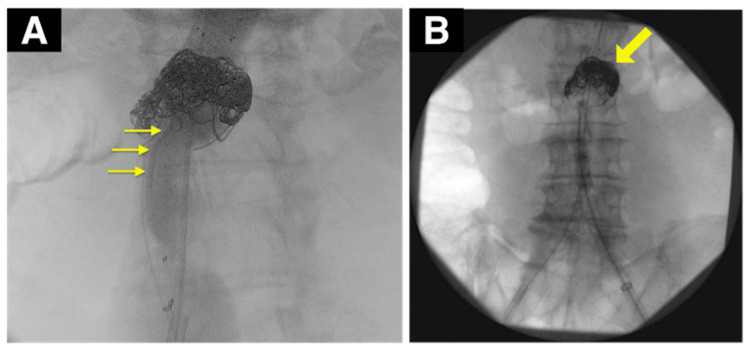
(A) Intraoperative angiogram: the tip of the catheter introduced from the left femoral artery was placed in an aneurysm sac of the proximal landing zone (yellow arrow). (B) Angiogram of the completed coil embolization is shown and no blood flow was confirmed inside the aneurysm sac by aortography.

Thus, the patient was diagnosed with a two-humped AAA with a short neck. However, conventional EVAR presented a potential risk for type Ia endoleak. While fenestration was an option, it could be complicated by the aorta’s severe calcification and internal thrombosis. The chimney method was also challenging because of the short distance between the renal arteries and the superior mesenteric artery. Therefore, we initially placed a catheter at the aneurysm neck. After stent graft deployment, if a type Ia endoleak was observed, we aimed to eliminate it by filling the aneurysm neck with coils. The surgery was conducted under general anesthesia, approaching through the right common femoral artery. Initially, we inserted a catheter (Angiographic Catheter, 4 Fr, 65 cm; Cordis, Inc., USA) from the left common femoral artery to the aneurysm for coiling (Figure 2A). After deploying the stent graft and performing angiography, we noted a type Ia endoleak. Hence, we advanced the catheter to the endoleak site at the aneurysm neck and used four 60 cm coils (POD Packing coil®; Penumbra, Inc., USA) for coiling (Figure 2B).

**Figure 3:**
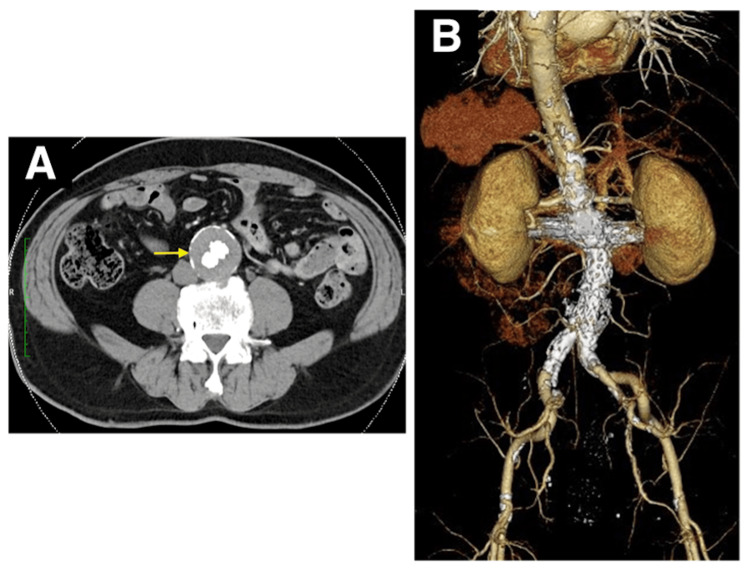
(A and B) Postoperative three-dimensional reconstruction computed tomography and computed tomography angiography showed favorable stent graft positions without type Ia endoleak.

**Figure 4:**
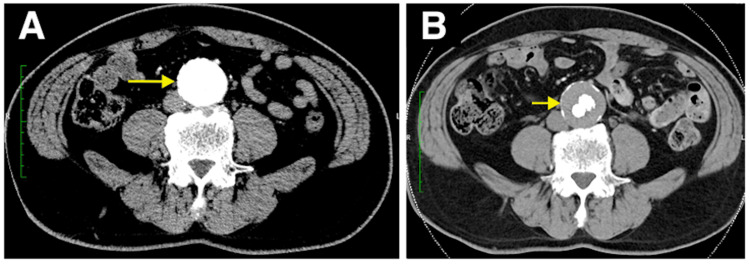
(A) Compared to the computed tomography angiography (CTA) before surgery. (B) CTA of the one-year follow-up after surgery showed sac shrinkage of 10mm.

In the final angiography, the endoleak was eliminated, concluding the surgery. On postoperative day 7, angiographic CT confirmed no endoleak (Figures 3A, 3B), and the patient was discharged. One year postoperatively, the endoleak did not recur, and the aneurysm diameter was reduced by 10 mm (Figures 4A, 4B).

## Discussion

**Figure 5:**
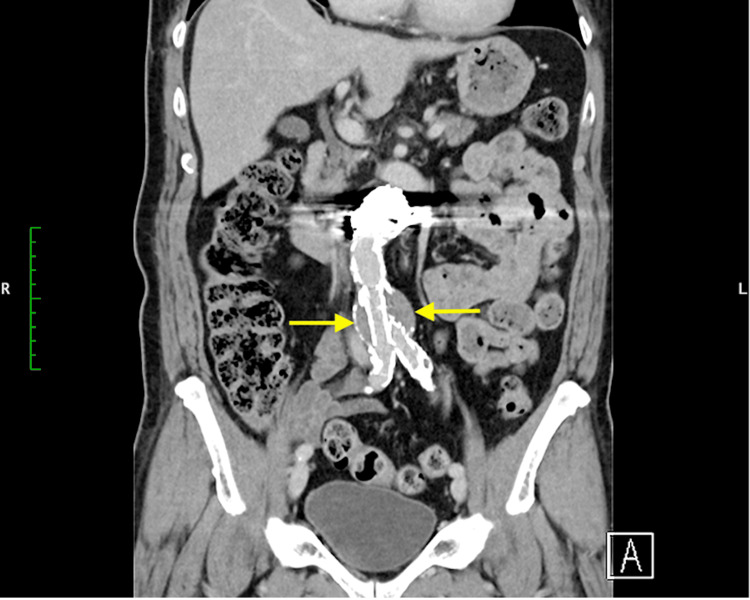
As observed in the CT image, it is possible to measure the maximum short diameter of the aneurysm as the coil is placed only in the neck.

According to the Vascular Surgery Annual Report in 2018, stent graft treatments for AAAs constitute 61.6%, thereby regarded as the standard therapeutic approach for AAAs [2]. However, challenging neck anatomy (short, conical, angulated, reverse conical, barrel, double barrel) is often encountered. Short-neck AAAs frequently lead to type Ia endoleak, which complicates the treatment [3]. In Japan, branched thoracoabdominal endovascular grafts remain commercially unavailable. Currently, treatments for short-neck AAAs include creating holes in the stent graft body aligned with abdominal branches by using the fenestration method (fenestrated endovascular stent grafts) or placing stents in the renal arteries by using the chimney method [4-5]. However, the fenestration method presents a risk for migration, which can lead to abdominal branch occlusion. This complication may be prevented by spot stenting in abdominal branches, but this approach is complex and not widely practiced [6]. Nevertheless, this method has potential issues, including gutter leaks and renal artery occlusion, and it cannot be used if the distance to the superior mesenteric artery is short [7]. Both fenestration and chimney methods demonstrate high secondary intervention rates [8]. In the present case, the fenestration method was not performed because of the aorta’s severe calcification, which could lead to endoleak, and the presence of multiple thrombosis within the abdominal aorta, which posed a risk for embolism. Owing to the short distance between the renal arteries and the superior mesenteric artery, the chimney method was not chosen. We initially placed a catheter within the aneurysm sac. After deploying the main body (Cook Medical, Bloomington, Indiana, USA), we noticed a type Ia endoleak. Despite placing an aortic cuff (W. L. Gore & Associates, Inc., Newark, DE), we could not control the endoleak. Therefore, we performed coil embolization at the aneurysm neck to eliminate the type Ia endoleak. This technique is convenient because it fills the gap between the stent graft body and the aneurysm neck with coils. The position of embolization can be determined by manipulating the catheter. Leaving the catheter in place allows for the addition of coils if a type Ia endoleak is observed in the final angiography. Using 60-cm-long coils reduces the number of coils needed. Postoperative CT may cause haloing, making aneurysm diameter measurement challenging; however, measuring the maximum short diameter of the aneurysm is possible, given that the coils are placed only at the neck (Figure 5).

A similar method involves injecting N-butyl-2-cyanoacrylate (NBCA) into the aneurysm [9]. However, considering that NBCA is a liquid, embolizing only the aneurysm neck is difficult, and the patient can be at risk for peripheral embolism. Although aneurysm sac embolization has been performed as a secondary intervention, selectively embolizing the aneurysm neck is challenging [10]. Traditionally, short-neck AAAs have been treated with OSR. However, patients with a history of prior conditions and those with a hostile abdomen face a high risk and potential postoperative complications. Our approach in this case may offer an effective alternative for treating short-neck AAAs.

## Conclusions

Patients with short-neck AAAs often face challenges when undergoing EVAR. In this report, we successfully treated an endoleak by performing coil embolization in the aneurysm neck. The initial catheter placement within the aneurysm facilitates a straightforward procedure. Therefore, this method may be advantageous for patients with short-neck AAAs.
